# Heterotopic Tubal Choriocarcinoma Coexistent with a Viable Intrauterine Pregnancy: A Case Report

**DOI:** 10.3390/medicina60081226

**Published:** 2024-07-29

**Authors:** Arnoldas Bartusevicius, Egle Bartuseviciene, Minginte Maseviciene, Arturas Sukovas, Ieva Birbalaite, Migle Karpaviciute

**Affiliations:** 1 Department of Obstetrics and Gynaecology, Lithuanian University of Health Sciences, 44307 Kaunas, Lithuania; egle.bartuseviciene@lsmu.lt (E.B.); arturas.sukovas@lsmu.lt (A.S.); 2 Department of Pathological Anatomy, Lithuanian University of Health Sciences, 44307 Kaunas, Lithuania; 3 Faculty of Medicine, Lithuanian University of Health Sciences, 44307 Kaunas, Lithuania; ieva.birbalaite@stud.lsmu.lt (I.B.); migle.karpaviciute@stud.lsmu.lt (M.K.)

**Keywords:** choriocarcinoma, pregnancy, tubal choriocarcinoma, ectopic pregnancy, heterotopic pregnancy

## Abstract

*Background and Objectives:* Choriocarcinoma is an aggressive oncological disease that manifests as trophoblast tissue proliferation. The vast majority of primary lesions affect the uterus, with primarily extrauterine lesions being a rarity. Choriocarcinoma with an ongoing pregnancy is extremely rare because fetuses usually do not survive the third trimester. *Case Report*: We present a case of heterotopic tubal choriocarcinoma coexisting with a viable intrauterine pregnancy. A 30-year-old, 39-week pregnant woman (gravida 2, para 2) came to our hospital complaining of acute upper abdominal pain. During routine prenatal screening in the first trimester, no pathological ultrasound findings were detected. Similar abdominal pain episodes had been recorded at 18, 27, and 32 weeks of gestation, when patient was hospitalized for examination and observation, but the cause of symptoms at that time of gestation remained unclear. The patient underwent an emergency caesarean section due to severe abdominal pain and fetal compromise. She delivered a live male infant. During the surgery, around 1000 mL of blood clots were evacuated, and the excision of the right fallopian tube and masses, as well as the control of significant blood loss was performed. Postoperative serum beta-hCG was elevated to 139 482 IU/L, while imaging studies showed no metastasis. The histological examination of the excised tissue samples confirmed a diagnosis of tubal choriocarcinoma. With a FIGO score of 8, the patient received three courses of the EP/EMA regimen. After more than a year, the patient showed no radiographic signs of distant metastasis and is now in complete remission. *Conclusions:* This case highlights the diagnostic complexity of such extremely rare scenarios. Even though such cases are rare, it demonstrates the necessity for improved diagnostic measures to enhance patient outcomes in similar clinical situations.

## 1. Introduction

Choriocarcinoma is an aggressive oncological disease that manifests as trophoblast tissue proliferation and acute vaginal bleeding. In Europe and North America, choriocarcinoma affects approximately 1 in 40,000 pregnancies, whereas in Southeast Asia and Japan, choriocarcinoma rates are higher, at 9.2 and 3.3 per 40,000 pregnancies, respectively [1]. According to data from the National Cancer Registry, during the period from 1993 to 2023, a retrospective analysis of medical records in Lithuania revealed the occurrence of 19 documented cases of choriocarcinoma originating from placental tissue (data from the National Cancer Institute Cancer Registry were obtained through personal communication). Choriocarcinoma is the most aggressive histologic type of gestational trophoblastic neoplasia (GTN) compared to invasive mole, placental site trophoblastic tumor (PSTT), and epithelioid trophoblastic tumor (ETT). This disease is characterized by high levels of human chorionic gonadotropin in the blood (hCG) and its stimulation effects, early vascular invasion, and widespread metastases to other organs, such as the lungs, liver, spleen, kidneys, and the brain [1].

Choriocarcinoma can be divided into gestational and non-gestational. The former consists of invasive hyperplastic and anaplastic trophoblastic tissue made up of atypical syncytiotrophoblast, cytotrophoblasts, and intermediate trophoblast components, whereas the latter originates from pluripotent germ cells [1]. It is crucial to evaluate patients’ previous obstetric history, because most choriocarcinomas follow complete moles (50%), less commonly spontaneous abortion (25%), normal term pregnancy (22.5%), or ectopic pregnancy (2.5%) [2]. The onset of choriocarcinomas may range from 4 weeks to 25 years after gestation or even after menopause [3,4]. Clinical presentation depends upon the extent of the disease and the location of metastases, but they usually present as acute bleeding from the uterus, menstrual irregularity, and pain and/or pressure sensation in the lower abdomen [5].

Choriocarcinoma with an ongoing pregnancy is extremely rare, because fetuses usually do not survive the third trimester [6]. There are only a few documented cases of choriocarcinoma with an ongoing (normal) pregnancy in the uterus. In most of these cases, the patient presents with advanced choriocarcinoma complications, such as intracranial metastasis, cerebral hemorrhage, and lung metastasis, as well as intra-abdominal and intrapleural hemorrhage [4,6,7,8,9,10,11,12,13]. The vast majority of primary lesions affect the uterus, with primarily extrauterine lesions being a rarity. Tubal choriocarcinoma accounts for only 4% of all reported cases of choriocarcinoma, and the incidence is reported to be 1.5/1,000,000 births [14].

In this report, we present an exceptionally rare case of heterotopic tubal choriocarcinoma coexisting with a viable intrauterine pregnancy and discuss diagnostic and management challenges.

## 2. Case Presentation

A 30-year-old patient (gravida 2, para 2) was admitted to the Lithuanian University of Health Sciences Hospital Kaunas Clinics, Department of Obstetrics and Gynecology at 39 weeks and 4 days of gestation complaining of severe acute upper abdominal pain. Similar acute abdominal pain episodes had been recorded at 18, 27, and 32 weeks of gestation, when the patient was hospitalized to the same referral university hospital for examination and observation. Her first pregnancy was a normal pregnancy four years before, and she had a natural childbirth of a healthy 4000 g baby. She had no significant medical or family history.

During routine prenatal screening in the first trimester of this pregnancy, no pathological ultrasound findings were detected. During the visit at 18 weeks of gestation, the woman complained of abdominal pain. An ultrasound scan revealed a small amount of free fluid in the abdominal cavity. Bloodwork showed a slightly elevated white blood cell count; however, the CRP was within the normal range. Furthermore, no signs of infection, peritonitis or urological conditions were observed. After a few days of careful monitoring, the woman was feeling well and was discharged. 

A similar acute upper abdominal pain episode also occurred at 27 weeks of gestation. She was hemodynamically stable, her body temperature was 36.1 C. The fetal heart rate was 142 bpm and the fetal cardiotocography (CTG) unremarkable. Blood work was within the normal range, except for lower hemoglobin 100 g/L. An abdominal ultrasound scan showed signs of ascites with free fluid being at up to 4.2 cm in some parts of the abdominal cavity. However, no other surgical conditions or causes for ascites were observed. The liver, gallbladder, common bile duct, and other abdominal organs showed no changes, except for the dilated collection system of the right kidney, with the ureter being 0.8 cm proximally. That prompted a differential diagnosis for kidney stones or obstructive pyelonephritis. However upon urological evaluation, an intervention was not clinically indicated. In view of her previous pain episodes and unclear cause of ascites, a multidisciplinary medical team decided to do a cancer marker workup, including Ca 125, Ca27.29, Ca19-9, and CEA, which were all within normal range. With no other causes for ascites, it was thought that the ascites were pregnancy-related, and no surgical intervention was indicated. The woman was again under observation for a few days. Later, she stated that her symptoms had gone, and she was discharged, with recommendations for outpatient observation. 

After 5 weeks, at 32 weeks of gestation, the woman was again brought to the emergency department experiencing severe generalized abdominal pain. Upon the objective examination, the patient was alert and oriented, hemodynamically stable. Upon palpation, the abdomen was soft to the touch, with no masses or rigidity, painful around the uterus. The peritoneal signs were negative. An abdominal ultrasound scan again revealed free fluid in the abdominal cavity. Blood work and urine analysis were unremarkable, the liver enzymes were within the normal range. The CTG showed no fetal compromise. Transvaginal ultrasound revealed no signs of premature labor or placental abruption. The woman received NSAID for pain management and after a day, when the abdominal pain had gone, she was discharged for outpatient observation. 

Now, at 39 weeks and 4 days of gestation, the pain is significantly more severe. At presentation, the woman was oriented, with a pulse of 97 bpm and blood pressure of 154/119 mm/Hg. The CTG showed decreased fetal heart rate, and moderate decelerations <80 bpm. No contractions or vaginal discharge were present. Bishop’s score of the cervix was 3, and the amniotic sac was intact. An emergency caesarean section (CS) was performed due to fetal compromise and severe abdominal pain. The woman delivered a male infant with an Apgar score of 5 and 8, with no congenital malformations.

**Figure 1:**
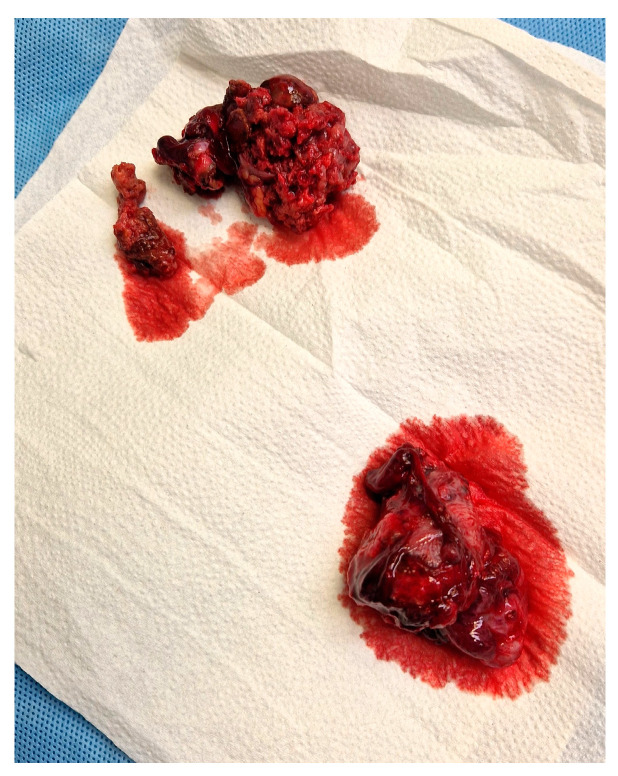
Right salpingectomy and tumor masses from the abdominal cavity.

During the surgery, the team encountered unexpected intra-abdominal findings warranting immediate attention. Firstly, around 1000 mL of blood clots were evacuated. Moreover, a heterogenic mass of about 6 cm was identified, adhered to the right ovary and fallopian tube, along with adhesions to the abdominal wall and caecum. The intestines displayed extensive deserosation, and signs of hyperstimulation were observed in the ovaries. The surgical intervention involved the excision of the right fallopian tube, along with the masses (Figure 1), as well as meticulous hemostasis to address the significant blood loss secondary to the evacuation of massive blood clots. Additionally, efforts were made to evaluate the extent of deserosation and restore the anatomical integrity to the intestinal serosa. These collective findings suggested a consideration for differential diagnoses of heterotopic pregnancy or neoplastic processes. Given the rarity of such findings during the CS, the fallopian tube, alongside the tumor masses and biopsies of the peritoneum were collected and sent for histological examination.

Following the caesarean section, the patient underwent postoperative imaging studies to evaluate potential metastatic spread. Chest, abdominal, and pelvic computed tomography (CT) scans revealed no evidence of metastasis. The postoperative serum beta-hCG was elevated to 139 482 IU/L, and it suggested the diagnosis of choriocarcinoma.

**Figure 2:**
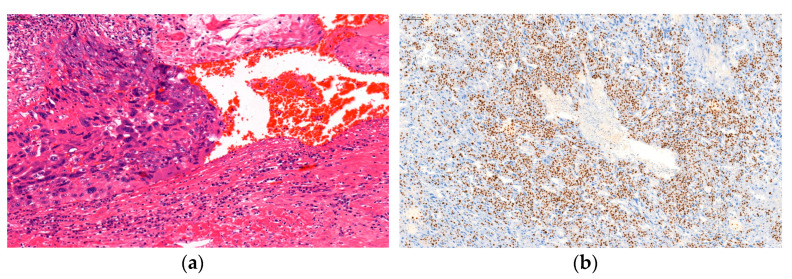
Nodular aggregates of atypical trophoblastic cells without chorionic villi. Brisk mitotic activity and lymphovascular invasion (H&E) (**a**); high proliferation is indicated by Ki-67 immunoreactivity (80–90%) (**b**).

Macroscopically, a nodular-shaped bloody 5 × 3, 3 × 3 cm size formation was observed at the ampullary part of the fallopian tube serosa, with overgrowth in the wall, at which the fallopian tube appeared thinned-walled. Histological examination revealed infiltrative, destructive, solid aggregates of highly atypical, predominantly mononuclear cytotrophoblasts and intermediate trophoblasts, rimmed by multinucleated syncytiotrophoblasts. Brisk mitotic activity, extensive necrosis, and lymphovascular invasion was found (Figure 2a). Although immunohistochemistry to confirm the diagnosis of choriocarcinoma is not obligatory, in some cases it can help to support the diagnosis. In our case, atypical trophoblast cells were immunoreactive for pan-CK and hCG and focally for inhibin. In addition, the Ki-67 proliferation index was high (80–90%) (Figure 2b). No chorionic villi, considered to be discordant with the diagnosis of choriocarcinoma, were found. Separate masses removed from the peritoneum histologically showed the same atypical trophoblastic cells. Macroscopical examination of the entire placenta, with 5 mm interval sections, as recommended, was performed but revealed no focal lesions for suspicion of intraplacental choriocarcinoma, and that was histologically confirmed. Thus, the final diagnosis of heterotopic tubal choriocarcinoma coexisting with a normal intrauterine pregnancy was confirmed.

The diagnosis of choriocarcinoma prompted multidisciplinary consultations involving oncologists and gynecological specialists to formulate an individualized treatment plan tailored to the patient’s specific clinical presentation and oncological needs. With a FIGO score of 8, the patient received three courses of the EP/EMA regimen (etoposide and cisplatin, with etoposide, methotrexate, and dactinomycin). After a total of three courses of the EP/EMA regimen, serum beta-hCG reduced significantly and has been negative during the follow-ups. After more than a year, the patient showed no radiographic signs of distant metastasis and is now in complete remission.

## 3. Discussion

Heterotopic pregnancy or simultaneous intrauterine and extrauterine gestation is a rare condition and usually occurs in women undergoing ovulation induction or in pregnancies following assisted reproduction techniques [15]. The reported incidence of heterotopic pregnancy is 0.6–2.5/10,000 pregnancies [16]. In natural cycles, as in our presented case, the incidence is still rare and unexpected. Moreover, cases of heterotopic pregnancy with normal ongoing intrauterine pregnancy and simultaneous tubal choriocarcinoma are extremely rarely reported.

Tubal choriocarcinoma is most often misdiagnosed with an ectopic pregnancy, since both share similar symptoms, including vaginal bleeding, amenorrhea, elevated serum beta-human chorionic gonadotropin (beta-hCG) levels, and pelvic pain [17]. Diagnostic errors occur because of its rarity and its presentation, mimicking a routine tubal pregnancy. Women usually undergo surgery for suspected tubal pregnancy, and only after histological examination of the specimen, together with highly elevated and non-decreasing beta-hCG levels, is the diagnosis of tubal choriocarcinoma confirmed, sometimes incidentally and unexpectedly [14,18,19,20,21]. 

In general, the serum beta-hCG of patients with tubal choriocarcinoma tends to abnormally elevate within a short time after amenorrhea, while that of patients with tubal ectopic pregnancy rarely exceeds 10,000 IU/L. In some cases, the serum hCG levels after surgery were not significantly reduced or even continuously increased [14,19,20]. In these cases, tubal choriocarcinoma, even a very rare entity, should be kept in mind. However, histological examination is the golden standard for diagnosing choriocarcinoma. It is crucial to carefully dissect and inspect the excised lesions during the operation. When no villous structures are visually observed in the excised lesions, it is strongly recommended to execute an intraoperative frozen section biopsy for the differential diagnosis between ectopic pregnancy and tubal choriocarcinoma [14].

The present case report deals with a rarer condition: tubal choriocarcinoma coexistent with an ongoing intrauterine pregnancy that extends to 39 weeks of gestation. We were able to find only one case reported describing heterotopic atypical trophoblasts mimicking ectopic choriocarcinoma coexistent with a viable intrauterine pregnancy [22]. In that case the woman underwent a laparoscopic right salpingectomy for a suspected ruptured ectopic pregnancy (lately diagnosed as non-neoplastic trophoblast of a very early conception mimicking ectopic choriocarcinoma) at 5 weeks of gestation, and one week later she was diagnosed with an intrauterine pregnancy of 6 weeks of gestation. This was a highly desired pregnancy and she was counselled for continuation of the pregnancy. However, the possibility of the earliest form of an intraplacental choriocarcinoma could not be excluded. Therefore, she was followed closely with serial beta-hCGs, which rose appropriately and then plateaued. She delivered a viable male infant at term via normal vaginal delivery [22].

In most documented cases of choriocarcinoma with an ongoing pregnancy in the uterus, the patient presented from 22 to 35 weeks of gestation (the mean gestational age at diagnosis was 26.5 weeks) with advanced choriocarcinoma metastasis in lungs (80–89%), vagina (17–30%), brain (3–29%), liver (10–11%), and with its related complications [4,7,8,9,10,11,12]. Consequently, in the majority of cases an emergency CS was performed prematurely.

Women with ongoing pregnancy do not demonstrate a typical clinical presentation of gestational trophoblastic neoplasia, or some of the symptoms, such as vaginal bleeding, uterine enlargement, or the enlargement of bilateral ovaries can be attributed to pregnancy [23]. The symptoms caused by metastases are not specific either [4]. However, diagnosis of choriocarcinoma without metastasis, coexistent with a live fetus, is a real medical challenge for gynecologists. Before the disease is symptomatic, the diagnosis can be established based on an abnormally high level of serum hCG [24]. Although elevated hCG levels are not pathognomonic for choriocarcinoma, a significantly elevated serum hCG, along with an appropriate history, is highly suggestive of choriocarcinoma [7,13]. Korevaar et al. determined in their study that the hCG concentration during pregnancy reaches the maximum at the end of the first trimester and then steadily decreases during the remaining gestation period, and is not higher than 74,719 IU/L after 25 weeks of gestation [24]. Other researchers have found a fall in the serum hCG concentration to a level of less than 1000 IU/L within the first 96 h after term deliveries, followed by a more gradual decrease during the next 2 weeks, until its complete disappearance [25]. Choriocarcinoma was considered in the differential diagnosis in our patient due to the high value of beta-hCG (139,482 IU/L), but only after a caesarean section. Imaging studies (ultrasound, MRI, and a CT scan, if needed) are recommended when diagnosing choriocarcinoma, but the neonate would be exposed to high levels of radiation [26].

We can now speculate that in our presented case, the abdominal pain episodes, which had been recorded at 18, 27, and 32 weeks of gestation, alongside with observed free fluid in the abdominal cavity on ultrasound scan, were the symptoms of either tubal abortion or rupture of the tube. However, the doctors at that time did not consider this diagnosis due to the rarity of heterotopic pregnancy. In addition, the spontaneous resolution of symptoms and the ongoing normal intrauterine pregnancy allowed the doctors to refrain from further investigations and encouraged the continuation of the pregnancy. After the second episode of upper abdominal pain at 27 weeks of gestation and unclear cause of ascites, the multidisciplinary medical team thought about oncological disease, but not about choriocarcinoma. Finally, the medical team discussed the possibility of laparocentesis, but the improvement in the woman’s condition at that time and the normal development of the pregnancy restrained doctors from performing an invasive examination during pregnancy. This suggests that the internal bleeding at that time of gestation was not as severe as described in the other case [7]. Nevertheless, the presence of the free fluid should have warranted more careful attention and further examination of the fluid itself. A diagnostic laparocentesis might have facilitated an earlier diagnosis, as well as serum hCG testing during pregnancy. Thus, although recommendations for the diagnosis of choriocarcinoma based on history, imaging studies, and serum hCG level are available, it is still challenging to properly and timely diagnose this pathology with a coexistent viable intrauterine pregnancy.

Since most patients with choriocarcinomas concurrent with pregnancy have been pregnant before (in our described case as well), it is difficult to determine whether the choriocarcinoma originated from the present pregnancy or their prior pregnancies. Therefore, for women with a history of childbirth, genetic analysis (STR DNA genotyping) of the specimen is required to distinguish between non-gestational choriocarcinoma and gestational choriocarcinoma and to determine which pregnancy was the origin [27]. However, genetic analysis is lacking in our presented case, as well as in most in scientific literature reported cases. Tubal choriocarcinoma may develop either by malignant transformation of a tubal pregnancy or can arise de novo without an ectopic pregnancy. Of the reported cases of tubal choriocarcinoma, the majority present as ectopic pregnancy.

Gestational choriocarcinoma is sensitive to chemotherapy drugs, so once diagnosed, chemotherapy is often the main treatment. Single-agent therapy may result in a cure in patients who are considered to have a low-risk disease based on the FIGO, and combination therapy offers a cure to over 80% of patients with a high-risk disease [23]. Therefore, the immediate postpartum multidrug chemotherapy was initiated on our patient, and ended with a satisfactory clinical effect. The women who are treated for extrauterine choriocarcinoma should receive effective contraception for at least one year after the completion of their treatment. After complete remission, the patient should be periodically checked for the rest of her life because of possible re-occurrence several years after the initial treatment. The follow-up marker is beta-hCG, and the monitorization of beta-hCG is the most useful diagnostic tool in case of tubal choriocarcinoma.

## 4. Conclusions

This case highlights the challenging diagnostic dilemma when there is heterotopic tubal choriocarcinoma coexistent with a viable intrauterine pregnancy. Typically, choriocarcinoma is suspected based on history, imaging studies, and highly elevated serum hCG levels, but it is still unclear how to properly and timely diagnose this pathology for patients with ongoing normal pregnancy due to the rarity of the disease and the lack of data and evidence. Our case emphasizes that choriocarcinoma should be considered in the differential diagnosis in any pregnant woman presenting with unexpected symptoms and unclear diagnosis.

## Data Availability

Data are contained within the article.
